# Assessing the impact of the reactivity of red brocket deer (*Mazama americana*) on training efficiency

**DOI:** 10.1371/journal.pone.0315488

**Published:** 2025-10-08

**Authors:** Lara Caveanha Gragnanello, Mariana Parra Cerezo, Cristiane Schilbach Pizzutto, Mateus José Rodrigues Paranhos da Costa

**Affiliations:** 1 Graduate Program in Animal Science, São Paulo State University (UNESP), School of Agricultural and Veterinary Sciences, Jaboticabal, São Paulo, Brazil; 2 Department of Animal Reproduction, Faculty of Veterinary Medicine and Animal Science, USP, São Paulo, São Paulo, Brazil; 3 Laboratory of Ecology and Evolution, Butantan Institute, São Paulo, São Paulo, Brazil; 4 Department of Animal Science, São Paulo State University (UNESP), School of Agricultural and Veterinary Sciences, Jaboticabal, São Paulo, Brazil; 5 The National Council for Scientific and Technological Development (CNPq), Brasília, Federal District, Brazil; Kerman University of Medical Sciences, IRAN, ISLAMIC REPUBLIC OF

## Abstract

The reactivity of wild animals has been studied due to its influence on *ex-situ* management and conservation strategies. However, there is insufficient research examining the impact of reactivity on training processes for veterinary handling procedures, which are essential for promoting the welfare of wild animals in conservation centers, zoos, and research institutions. This study aimed to evaluate the influence of reactivity on training for veterinary procedures in red brocket deer (*Mazama americana*). The reactivity of twelve red brocket deer were measured by recording their behavior in the presence of an unfamiliar person and during routine handling procedures. Subsequently, the deer were subjected to habituation and operant conditioning (employing positive reinforcement) for veterinary handling procedures. There were individual differences in the habituation process. Nine deer, which voluntarily approached the unknown person and showed calmness during handling procedures, progressed to the second phase of the study. The remaining three stayed in the habituation stage throughout the study and exhibited positive values in dimension one of Multiple Correspondence Analysis (MCA), indicating that they required more habituation sessions. Only five deer that participated in operant conditioning learned all the proposed commands, with four demonstrating very low reactivity in the behavioral tests. The overall success rate was 41.66%, indicating that only five of the twelve deer completed the learning process. We concluded that the most reactive deer, characterized as restless and agitated, performed poorly in habituation and operant conditioning, while less reactive deer, which were calmer and exhibited minimal movement, learned more commands. This study provides insights that can contribute to developing management strategies for cervids, facilitating the implementation of more personalized training approaches, and promoting animal welfare and handlers’ safety.

## Introduction

Temperament refers to the unique characteristics that determine consistent patterns of feelings, thoughts, and behaviors over time and across different conditions within individuals of the same species [1–3]. Research identifies temperament as a multifaceted trait that encompasses various dimensions, including avoidance [4], fear, reactivity [5], learning capacity [6], and responses to human interactions [7,8]. However, many of these studies typically employ assessment methods focusing on only one or a few aspects of temperament. Understanding temperament is essential for grasping the interactions between individuals and their environment [9], particularly when implementing appropriate management practices and promoting animal welfare [10].

Reactivity is a component of temperament, encompassing the intensity, latency, and speed of an individual’s behavioral responses to a stimulus [11]. In deer, high reactivity often complicates human approaches, posing significant challenges for effective interactions [12]. While such behaviors may confer adaptive advantages in natural environment by reducing the risk of predation [13,14], they also create obstacles in management settings. For example, heightened reactivity can hinder veterinarians’ ability to safely approach animals, thereby delaying early disease diagnosis and treatment [12].

This issue gains particular importance when red brocket deer are involved in *ex-situ* conservation programs. This species can grow up to 65 cm in height and 145 cm in length and have a weight of between 30 and 40 kg, exhibits a crepuscular activity pattern, is a solitary species [15], and occurs from northern Argentina to the Panama Canal [16]. According to the International Union for Conservation of Nature (IUCN) Red List of Threatened Species classification, *M. americana* is categorized as “Data Deficient”, adopted which is “…when there is inadequate information to make a direct or indirect assessment of its extinction risk based on its distribution and/or population status...” [16], highlighting the importance of studying its behavior to aid conservation efforts.

According to Duarte et al. [17], physical restraint of *M. americana* is discouraged because of the power of the hind legs, which poses a risk of injury to personnel. Consequently, chemical restraint often becomes the only viable option. The application of sedatives through dart guns may cause injuries to the animals [18], leading to physiological changes that can affect their behavior for several days [9], further complicating their management in human care.

Considering these challenges, training through the operant conditioning technique is a valuable tool in conservation centers and zoological institutions [19]. This learning process can shape behavior through positive reinforcement, establishing associations between specific actions and their outcomes [20]. Results from previous studies have confirmed the value of operant conditioning in various species within zoo settings. They demonstrated, for example, the effectiveness of operant conditioning with positive reinforcement in eliciting voluntary movements in giant pandas [21], successfully training giraffes [22,23] and tigers [24] for veterinary procedures and teaching chimpanzees to use a nebulizer [25] and to operate heart rate monitoring sensors [26].

However, the successful implementation of operat conditioning techniques depends heavily on the training and qualifications of the personnel involved. [18,27]. It requires skilled keepers who possess a thorough understanding of the animals’ individual needs, as well as substantial resources, particularly time and effort from technicians [18]. Therefore, investing in specialized training for those working directly with the animals is essential to ensure the effectiveness and welfare outcomes of such conditioning procedures.

Operant conditioning is essential for developing management routines [28,29], as it encourages animals to respond to commands and interact positively with caregivers and veterinarians [30–33], besides providing animals with a sense of control over their environment [34]. By utilizing positive reinforcement, this approach not only significantly enhances the standard of life of the animals but also reduces the necessity for physical and chemical restraints during management [35]. Despite its advantages, the limitations highlight the need for specialized training and resources, which may limit the accessibility of these practices across different species [27].

Despite some advancements, gaps still exist in understanding how the individual reaction of deer influences their responses to operant conditioning. The relationship between reactivity and learning remains underexplored in the *Mazama americana*, which is an ideal species for studying these aspects due to its natural high sensitivity to human presence. This study aims to clarify how individual differences impact the success and effectiveness of training of red brocket deer. The findings from this study have the potential to inform more effective management practices, enhance the quality of life for *ex-situ* deer, and contribute to conservation strategies that recognize the importance of their reactivity on adaptation and survival.

## Materials and methods

This study was approved by the Ethics Committee on the Use of Animals of the São Paulo State University (UNESP), School of Agricultural and Veterinary Sciences, Jaboticabal, SP, Brazil (protocol number 710/21).

### Animals and location

The study was conducted with 12 red brocket deer (*Mazama americana*) at the Deer Research and Conservation Center (NUPECCE, registered in SisGen, number AC0AA2A) of the São Paulo State University (UNESP), School of Agricultural and Veterinary Sciences, in Jaboticabal-SP, Brazil. The subjects included eight males and four females of various ages, all born and raised at NUPECCE. Table 1 shows detailed information about each animal.

**Table 1:** Name, sex, and age of the deer that took part of this study.

NAME	SEX	AGE(YEARS)
**ARIEL**	Male	3
**CHICO**	Male	11
**CORAGEM**	Male	3
**EVE**	Female	7
**FELIPE**	Male	4
**GUTA**	Female	9
**HULK**	Male	8
**KIDA**	Female	1
**LAMPIÃO**	Male	5
**LUCIANA**	Female	10
**MARQUINHOS**	Male	9
**TEMER**	Male	4

At NUPECE, the red brocket deer are housed in a large shed with screened openings approximately 1 meter in height on the upper part of all four walls. This design promotes better airflow and allows for natural light and sunlight to enter. The shed contains individual enclosures, each ranging from 8 to 12 m^2^, separated by masonry walls that reach about one-third of the height of the structure. Each enclosure has a wooden door facing a central corridor, which connects the pens to the outdoor paddocks and the handling room. The handling room houses the management and veterinary treatment area, which is used for the temporary restraint of animals during procedures, ensuring safety for both the animals and the personnel.

### Reactivity assessment

Four tests were conducted prior to the start of training procedures to assess the deer’s reactivity, during the period from May to September 2021. Two researchers carried out the tests, with one having no prior contact with the animals (unfamiliar). Assessments occurred in three distinct contexts: (1) with the deer in their home stalls, measuring their reactivity when in the presence of an unfamiliar person; (2) when driving deer to the handling box, scoring their reactivity while moving them; and (3) when the deer was within the handling box, assessing their reactivity both during confinement and upon touch. The specifics of each context are detailed below.

#### Reactivity test in the presence of an unfamiliar person.

This test followed the methodology established by Waiblinger et al. [36], with necessary adaptations to fit the study’s framework. The animal was alone in the stall. An unfamiliar person slowly opened the stall door and positioned herself within the animal’s line of sight. She remained at the door for 2 minutes without making any movement, allowing the deer to familiarize herself with her presence. In the next 10 minutes, we recorded whether the animal approached the unfamiliar person or not (approach: 1 = yes or 0 = no). After 12 minutes, the unfamiliar person slowly approached the deer, extending her right arm to touch its neck gently. The animals were then scored based on their restlessness, as described in Table 2.

**Table 2:** Restlessness scores assigned to the red brocket deer (Mazama americana) when approached by an unfamiliar person.

Score	Restlessness	Description
**1**	Extremely calm	Deer allows being touched by an unfamiliar person without attempting to escape.
**2**	Calm	Deer remains calm but does not allow being touched, moves around the enclosure, and allows being touched but shows some reluctance.
**3**	Slightly restless	Deer shows moderate agitation and avoids the approach of the unfamiliar person but does not exhibit aggressive behavior or sudden movement.
**4**	Restless	Deer runs back and forth in the enclosure when approached by the unfamiliar person but does not attempt to jump over the walls or hit the ground with its front paw to escape.
**5**	Extremely restless	Deer attacks the unfamiliar person or runs back and forth in the stall, frequently looking up and attempting to jump against the walls.

#### Reactivity tests during handling procedures.

On a different day from the previously described tests, we assessed the deer’s reactivity during the transfer from their home stalls to the handling box, which had an average length of 10 meters. During this test, we recorded whether the deer jumped (jump over 1 meter high) or not (jump: 1 = yes or 0 = no) while being driven from the stalls to the handling box. The handling box measures 108 cm x 82 cm x 38 cm and features two guillotine-style gates on the shorter sides that open by pulling the lid upwards. Additionally, it has eight holes on the sides and two guillotine-style windows on the top (S1 Fig).

After each deer entered the box, the gates were closed, and the following tests were conducted 1) the trainer opened one of the upper windows and gently touched its hindquarters and, based on their responses, the trainer assigned one of the movement scores described in Table 3; and then the trainer touched on the backs of the deer again and scores its body posture (posture: standing = 1, kneeling = 2, or 3 = lying in a sternal position).

**Table 3:** Movement scores assigned to the red brocket deer (Mazama americana) when kept in the handling box.

Score	Movement	Description
**1**	No movement	Stand still without the lower limbs.
**2**	Minimal movement	Lacking vigor and remaining stationary for more than half of the interaction time.
**3**	Movement	With vigor and remaining stationary for more than half of the interaction time.
**4**	Frequent and vigorous movement	Characterized by back-and-forth movement within the box for more than half of the interaction time.
**5**	Excessive movement	Including jumping and attempts to escape by pushing against the box with its hindquarters.

### Learning processes

The learning process comprised habituation and operant conditioning, conducted through daily sessions from 9 A.M. to 2 P.M., incorporating at least one rest day weekly. Furthermore, all sessions underwent recordings by video for documentation and analytical purposes. The first phase emphasized habituating the deer to the trainer and promoting positive interactions (see S2 Fig). In the subsequent phase, the trainers implemented operant conditioning techniques to train the deer in veterinary management practices. Throughout the learning process, the materials utilized included a *target* to guide the desired movements and actions, food rewards (pieces of banana), and secondary rewards that included verbal praise (e.g., ‘well done’) and a clicker to reinforce positive behaviors.

Each habituation session lasted approximately 10 minutes, beginning with the trainer opening the stall door. During the session, the trainer actively interacted with the deer by calling it by name and engaging in conversation. Upon entering the stall, she positioned a chair and set down a food container filled with feed while holding a bowl of banana pieces. After approximately eight minutes, she offered the banana pieces to the deer. If a deer approached, she extended the bananas by hand; if not, she tossed them nearby. This procedure was realized until the deer demonstrated calm behavior (waiting at the door when called, voluntarily approaching the handler, and not showing sudden or startled movements) or reduced escape responses (not looking upward or attempting to jump the wall). Over time, the trainer began to enter the stall, moving slowly among the animals, calling to it, and showing the banana pieces.

The second phase of the learning program emphasized operant conditioning (see S2B Fig), based on the principle of continuous reinforcement, rewarding each correct response to a command. She repeated the deer’s name three times before entering the stall to establish familiarity. This phase began with the trainer encouraging the deer to approach her calmly as she entered the stall, offering a piece of banana upon her arrival; if the deer hesitated, she would bring the fruit closer to it.

After the deer was engaged, the trainer positioned herself in the corner of the stall and initiated the conditioning process by issuing the command “Come” and showing the banana. Employing a trace-conditioning method, the trainer pressed a clicker upon receiving a correct response from the deer, immediately followed by a fruit reward. Success was indicated by the deer approaching in response to the voice command without requiring a visual cue of the reward.

The learning progress of each deer modulated the application of operant conditioning commands. For instance, a deer that mastered the command “Belly” had previously successfully performed the commands “Come” and “Snout.” Each operant conditioning session began with commands the deer had already learned and was consistently concluded with the command “Over”, at which point a container of feed placed inside the stall was topped with a larger quantity of banana pieces as a reward. Table 4 shows detailed information regarding the commands utilized during training.

**Table 4:** Commands applied during the operant conditioning process, respective objectives, and illustrations displaying the practical application of each command.

Commands	Objectives
Come + deer name	To call the deer and initiate the operant conditioning process.
Snout	To position the deer in a way that facilitates operant conditioning.
Belly	To desensitize the deer to the touch of the *target*.
*Spray*	To desensitize the deer to the sound of the *spray*.
Back	To desensitize the deer to touch on the back and to administer medication (antiflea treatment, acaricide, and pour-on solutions).
Over	To finalize the conditioning process. The rewards must be highly appealing to ensure the deer does not associate the command “over” with a negative experience.

Due to the individual differences among the animals when reacting to the trainer’s presence, we adapted the way to approach and which movements and proximity to adopt for each deer during habituation and operant conditioning procedures. For example, Luciana accepted only when the trainer approached from her right side, while Eve and Kida did not allow the trainer enter the enclosure before completing the habituation procedure.

### Statistical analysis

Statistical analyses were conducted using R software, version 4.3.3. The Cronbach’s alpha coefficients were estimated to assess the internal consistency reliability of the behavioral variables obtained in the five tests applied to determine deer reactivity. The estimates of Cramer’s V coefficients were used to measure the pairwise association between the behavioral variables.

The associations between the behavioral variables used to evaluate deer reactivity, including the scores of approaches, restlessness, posture, and movement (S1 Table), were estimated by performing a multiple correspondence analysis (MCA). A linear regression analysis was used to evaluate the relationship between the coordinates of each deer obtained in the MCA and the number of sessions required for deer habituation. MCA coordinates were treated as predictor variables in the regression analysis.

## Results

The Cronbach’s alpha coefficients (0.74 and 0.79, raw standardized data, respectively) indicate an internal consistency between the variables recorded. The contribution of each variable was determined by identifying the changes in Cronbach’s alpha coefficients when each one was removed individually. The results showed that ‘jump’ and ‘restlessness’ offered the highest contributions to the overall consistency, while ‘posture’, the lowest. These support that most of the variables represent complementary measures to assess deer reactivity. The low contribution of the ‘posture’ can be explained in future studies.

Additionally, the results of Cramér’s V association (Table 5) show that most of the behavioral variables are not independent, with ‘jump’ showing a moderate association with ‘movement’ and ‘approach’, and ‘restlessness’ showing a high association with ‘movement’ and ‘posture’, and a perfect association with ‘jump’ and ‘approach’. Despite this dependence, these variables were included in the MCA because they provided complementary practical information about deer behavior during handling routines.

**Table 5:** Cramér’s V coefficients between the behavioral variables.

Behavioral variable		Cramér´s V coefficients
Jump	Movement	0.654
Jump	Posture	0.408
Jump	Approach	0.500
Jump	Restlessness	1.00
Movement	Posture	0.317
Movement	Approach	0.462
Movement	Restlessness	0.775
Posture	Approach	0.000
Posture	Restlessness	0.816
Approach	Restlessness	1.00

Table 6 shows the frequencies of the scores assigned for each of the five variables used to define the reactivity of the 12 red brocket deer engaged in this study. These frequencies formed the basis for the Multiple Correspondence Analysis (MCA).

**Table 6:** Frequencies of scores assigned for each behavioral variable of five reactivity tests.

Reactivity test	Behavioral category/ Score	Frequency
**Approach**	Approached/ 1	4
	Did not approach/ 0	8
**Restlessness**	Extremely calm/1	4
	Calm/ 2	4
	Slightly restless/ 3	1
	Restless/ 4	2
	Extremely restless/ 5	1
**Jump**	Jumped/ 1	4
	Did not Jump/ 0	8
**Posture**	Standing/1	9
	Lying/ 3	3
**Movement**	No movement/ 1	7
	Minimal movement/ 2	3
	Excessive movement/ 5	2

**Fig 1:**
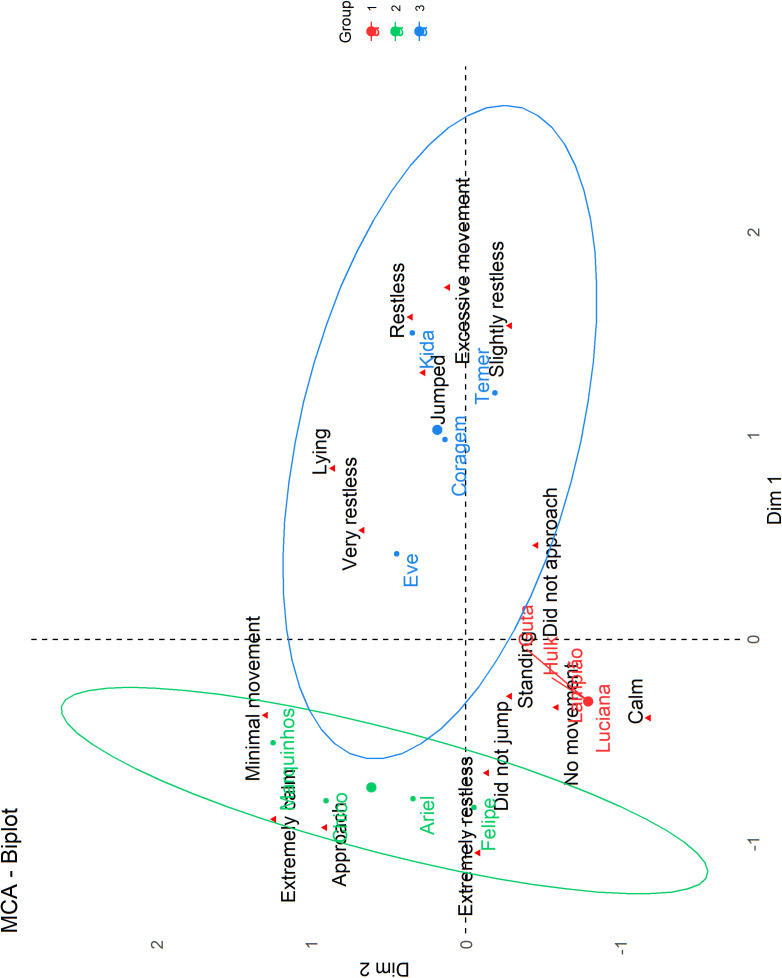
Two-dimensional correspondence plot using MCA, showing the spatial distributions of deer and behavioral categories expressed during the reactivity tests.

The first three dimensions of MCA explained 76,5% of the data variation (34.3% and 24.2%, and 17,9%, for the first, second and third dimension, respectively). Table 7 shows the deer’s coordinates in dimensions 1, 2 and 3 of the MCA, with negative values representing behavioral expressions that indicate lower reactivity and vice versa. Fig 1 shows the spatial distributions of deer and behavioral categories expressed during each reactivity test considering dimensions 1 and 2.

**Table 7:** Multiple correspondence analysis coordinates for each red brocket deer (Mazama americana) in the first two dimensions based on the results of reactivity tests conducted before training (in increasing order in dimension 1).

Animal	Dimension 1	Dimension 2	Dimension 3
Chico	−0,80	0,80	0.21
Ariel	−0,79	0,27	−0.19
Felipe	−0,79	0,27	−0.19
Marquinhos	−0,53	1,15	−0.27
Hulk	−0,29	−0,82	−0.05
Guta	−0,29	−0,82	−0.05
Lampião	−0,29	−0,82	−0.05
Luciana	−0,29	−0,82	−0.05
Eve	0,41	0,41	1.42
Coragem	0,98	0,16	−0.81
Temer	1,21	−0,16	0.72
Kida	1,50	0,38	−0.67

Four deer, Kida, Coragem, Eve and Temer, characterized by positive MCA coordinates, were situated farther from the deer and associated with behaviors such as jumping and restlessness. On the other way, Ariel, Chico, Marquinhos and Felipe displayed more positive behaviors, including approaching closely and remaining very calm and Luciana, Guta, Hulk and Lampião exhibit associations with intermediate behaviors between the two extremes, such as not approaching closely and remaining calm with no movement.

The associations among the coordinates of each deer in the first and second dimensions obtained in the MCA and the number of sessions required to perform conditioning is defined in the following linear regression equation:

Number of sessions for habituation = 22.33 + 23.32(CD1) + 10.2599(CD2), with r^2^ = 0.596, which indicates that approximately 59.6% of the variance in the number of habituation sessions can be explained by the coordinates dimensions obtained in 1 (CD1) and 2 (CD2) of the MCA. The equation shows that dimension 1 has a greater impact on the number of sessions required for deer habituation than dimension 2, with p values = 0.0076 and 0.238, respectively. Although the r^2^ value indicates a satisfactory level of explanation, it also suggests that factors other than the results of the reactivity assessment may influence the number of sessions required for deer habituation. Tests were carried out using the Mann-Whitney test to assess whether sex had any effect on the MCA coordinates and no statistically significant effects were found, indicating that the dimensions are independent of sex. D1 (p = 0.19) or D2 (p = 0.80).

**Fig 2:**
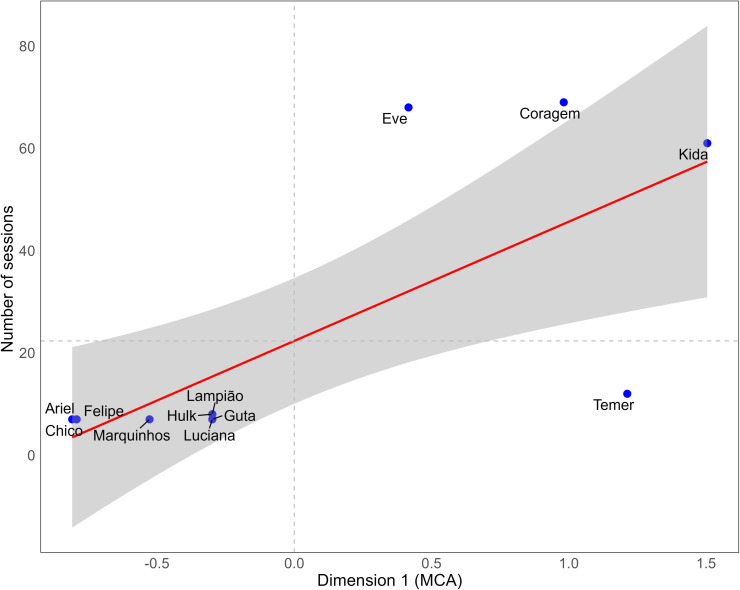
Linear regression among each red brocket deer (Mazama americana) of dimension 1 and the number of sessions required to habituate them to the trainer.

Visual analysis of the distribution of points representing each of the deer (Fig 2) shows that the deer that required the fewest sessions for habituation (Ariel, Chico, Felipe, Guta, Hulk, Lampião, Luciana and Marquinhos) were those whose coordinates in dimension 1 (x-axis) indicated that they were the least reactive deer. On the other hand, the most reactive deer (Kida, Coragem, Eve) required more sessions to achieve successful habituation. The coordinates of dimension 2 did not show a significant relationship with the number of habituation sessions.

The number of habituation sessions changed according to the deer reactivity. For instance, Ariel, Chico, Felipe, Guta, Luciana, and Marquinhos, classified as less reactive and showing positive behaviours, required only seven habituation sessions. On the other hand, animals with intermediate reactivity (Hulk, Lampião, and Temer) required 8, 8, and 12 sessions, respectively, and the more reactive ones (Coragem, Eve, and Kida) remained in the habituation stage throughout the study, participating in 69, 68, and 61 habituation sessions, respectively.

**Fig 3:**
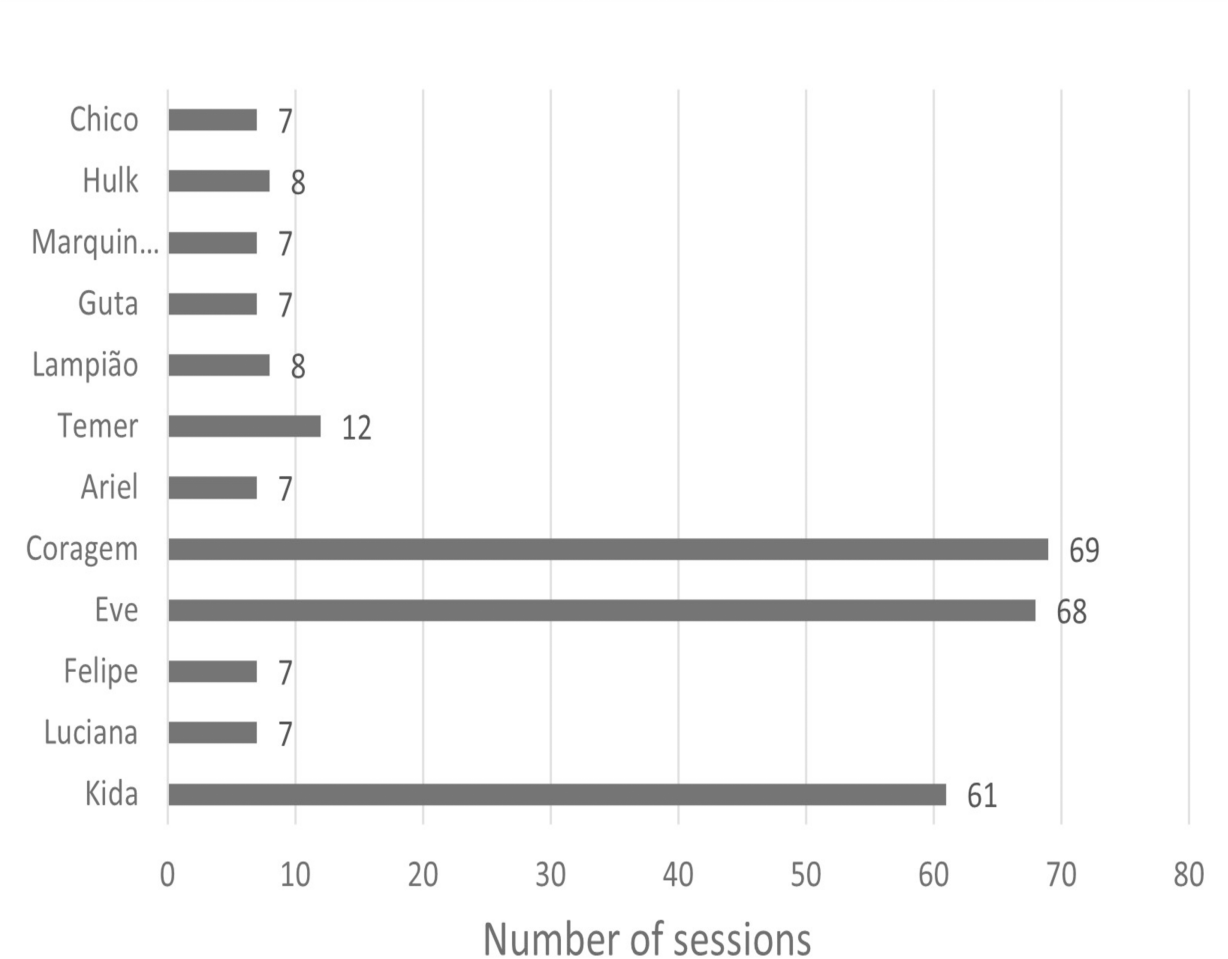
Number of sessions needed for each deer to become habituate to the trainer’s presence in the enclosure.

**Fig 4:**
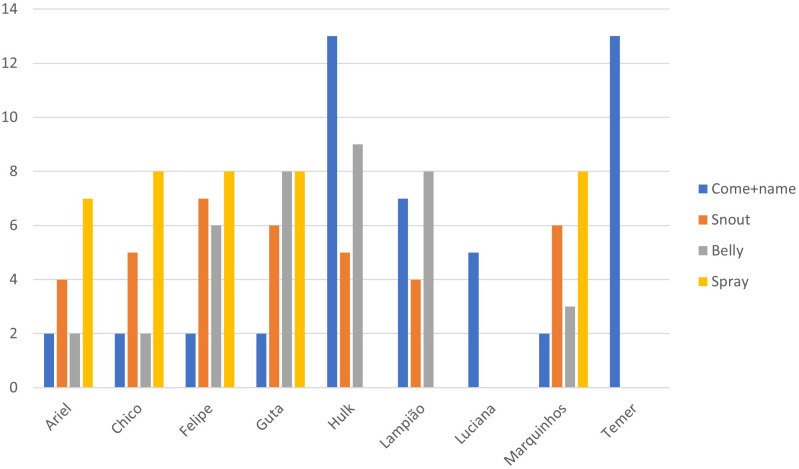
Number of operant conditioning sessions required for each red brocket deer (n = 9) to learn the commands for veterinary procedures. The bars indicate the commands learned.

After the habituation phase, 9 of the 12 deer moved on to the operant conditioning process, training for the commands: ‘come + name’, ‘snout’, ‘belly’, and ‘spray’. Fig 3 highlights the habituation progress of each deer and Fig 4 shows the number of operant conditioning sessions for each deer to learn the commands for veterinary care. The ‘back’ command was not included in the Fig 4 because no deer learned it.

## Discussion

Comprehending the temperament of each animal is crucial for optimizing operant conditioning techniques [37] which in turn can enhance training success rates [38]. Results from previous studies indicate that bolder deer are inclined to utilize anthropomorphized habitats more efficiently [39] and exhibit reduced migration patterns due to easier habituation [8]. This relationship suggests that a deer exhibiting bold behaviors is associated with a higher degree of adaptability to various environments and tolerance for changes, which, in turn, may facilitate the learning processes. The results of this study corroborate these reports, indicating the need to tailor training strategies to each individual based on their differences in reactivity. Thus, if trainers understand the degree of reactivity of each deer, they can adjust their approach when entering the enclosure, approaching the animal and defining how to move around it. This will promote a more effective and positive connection with the deer, making the habituation and operant conditioning with positive reinforcement process more efficient.

Four deer (Ariel, Chico, Felipe, and Marquinhos) showed a strong association with positive behavioral categories (such as calm and no movement) and required fewer (only 7) sessions for habituation. Among the nine deer that participated in the operant conditioning process, only the four mentioned, along with Guta, managed to learn the “*spray*” command, resulting in a success rate of 41.66% in learning most of the trained commands.

Previous results indicate that animals with higher curiosity and exploratory behavior generally demonstrate better adaptation to stressful situations and a greater willingness to participate in training [40], as well as superior behavioral flexibility [41]. Similar responses have been observed in *Cervus canadensis*, where bolder individuals were more responsive and learned more quickly [7].

In contrast, restless animals often face difficulties with changes and adaptation [1,12]. These findings reflect the slower progression of the deer in learning processes [6]. Our results corroborate this statement, showing that deer with the most negative indices took longer to habituate. The three deer (Kida, Eve, and Coragem), which had the lowest rankings, remained in the initial phase of the study, requiring more than 60 habituation sessions, without success. Such results are aligned with those reported by Huang et al., [13], as these three deer also jumped during the reactivity test handling procedures and displayed more intense movement while in the handling box. Similar behavior has been documented in forest musk deer (*Moschus berezovskii*) [42] and alpine musk deer (*Moschus chrysogaster*) [13]. Furthermore, comparable findings were reported in horses [43], showing that animals displaying negative behaviors such as fleeing, jumping during handling, intense movements, and agitation tend to show the poorest learning performances.

The other four deer (Guta, Hulk, Lampião and Luciana), which exhibited intermediate reactivity degree, advanced to the second phase of operant conditioning, but not all mastered every command except for Guta. Notably, Guta was an extremely aggressive female, yet she excelled at learning. In contrast, the other three deer exhibited more fearful behaviors toward the trainer. However, such response was not strong enough to inhibit the learning process, and they learned commands throughout the training process.

The learning process in deer displayed significant individual variation, as each animal progressed at its own pace, reflecting their distinct behavioral traits. This finding aligns with other studies suggesting that differences in responses to behavioral assessments are influenced by the unique characteristics of the animals [7]. Furthermore, it supports the idea that reactivity traits are directly related to success in operant conditioning [44]. However, it is essential to acknowledge that factors such as the training environment [33,45], prior negative experiences with humans [46], the trainer’s approach [47] and the consideration of individual animal preferences [48] also play critical roles in this process. The training setting and the nature of the trainer-animal interactions can substantially influence learning outcomes. In the present work we can observe that 40.4%, in the linear regression test, and 23.5% in the MCA were not related to deer reactivity (Figs 1 and 2). Therefore, recognizing these variations not only enhances our understanding of training processes but also informs better management practices aimed at promoting animal welfare in conservation contexts.

Although this study provides valuable insights, certain limitations should be acknowledged. The relatively small sample size may restrict the generalizability of the findings across the species. Additionally, individual variability in reactivity, behavior, and factors such as prior human interaction could influence the outcomes, indicating that personalized approaches might be necessary in practical applications. Importantly, we did not assess the influence of age, which can significantly affect behavioral responses and reactivity. Future research should prioritize this variable to better understand its impact. Despite these limitations, we recommend the training procedure used here, as it demonstrated effectiveness in improving animal cooperation and reducing stress during handling. Further studies could explore increasing the number of sessions for more reactive individuals, developing tailored protocols based on behavioral patterns, and explicitly examining the role of age to enhance the efficacy and applicability of these methods.

## Conclusions

The results of this study revealed that less reactive deer demonstrated a greater learning capacity, executing most of the applied commands accurately. On the other hand, the more reactive and fearful individuals exhibited a slower progression in mastering those commands. However, other variables may also influence learning processes, emphasizing the inherent complexity of animal learning. These findings emphasize the importance of individuality in the learning process of red brocket deer, particularly concerning reactivity. The analyzed behaviors, such as voluntary approach, jump, movement and restlessness in stressful situations were crucial for determining the effectiveness of training. Thus, reactivity plays a vital role in how animals respond to training, suggesting that management strategies should be tailored to individual behavioral traits to optimize conditioning outcomes.

## Supporting information

S1 Fig Dimensions of the handling box used for physically restraining red brocket deer (*Mazama americana*) during veterinary procedures and reactivity tests for this study. (DOCX)

S2 Fig Illustration of the interaction between a red brocket deer (*Mazama americana*) and the trainer during the learning process. (A) Positive interaction between the trainer and the deer ‘Chico’, during the habituation process. (B) Deer ‘Ariel’ is subjected to a desensitization process to spray noise as part of the training protocol.
(DOCX)

S1 Table Description and scales of behavioural variables. (DOCX)
