# Supplementary material for: Assessing the impact of the reactivity of red brocket deer (Mazama americana) on training efficiency
Source: PLoS One. 2025 Oct 8;20(10):e0315488. doi: 10.1371/journal.pone.0315488 (PMC12507295; doi:10.1371/journal.pone.0315488)
Supplement: S1 Fig — (DOCX) [file pone.0315488.s001.docx]

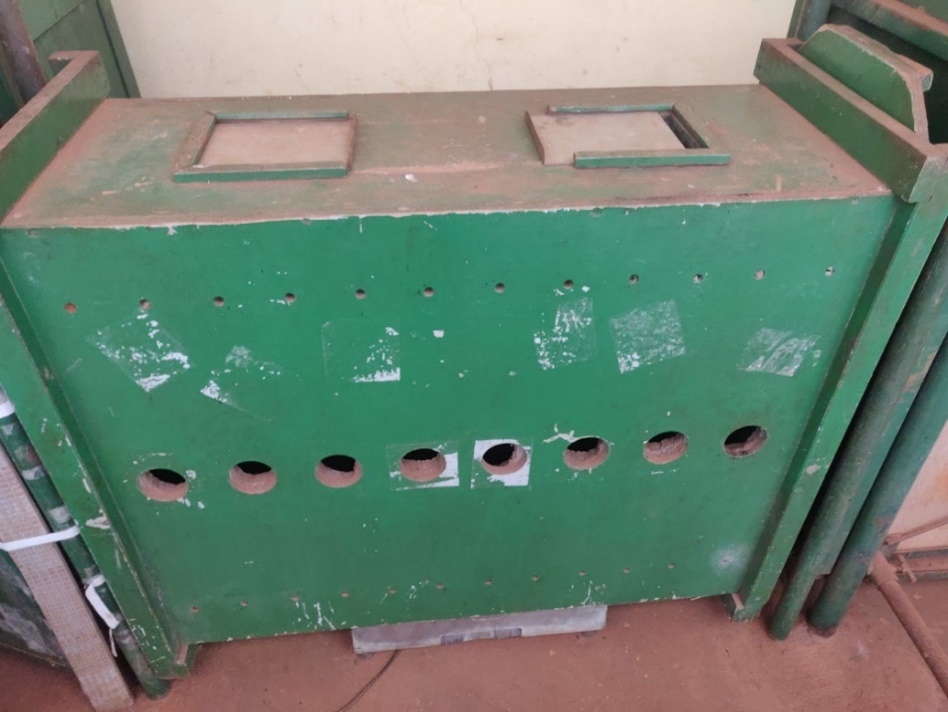


82 cm

108 cm

38 cm

**S1 Fig.** **Dimensions of the handling box used for physically restraining red brocket deer (Mazama americana) during veterinary procedures and reactivity tests for this study.**
