# Supplementary material for: Assessing the impact of the reactivity of red brocket deer (Mazama americana) on training efficiency
Source: PLoS One. 2025 Oct 8;20(10):e0315488. doi: 10.1371/journal.pone.0315488 (PMC12507295; doi:10.1371/journal.pone.0315488)
Supplement: S2 Fig — (A) Positive interaction between the trainer and the deer ‘Chico’, during the habituation process. (B) Deer ‘Ariel’ is subjected to a desensitization process to spray noise as part of the training protocol. (DOCX) [file pone.0315488.s002.docx]

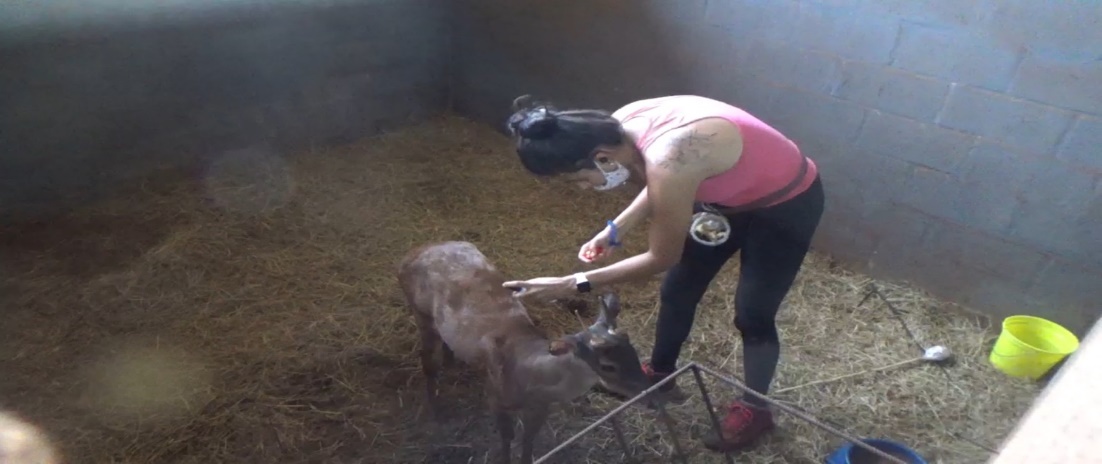

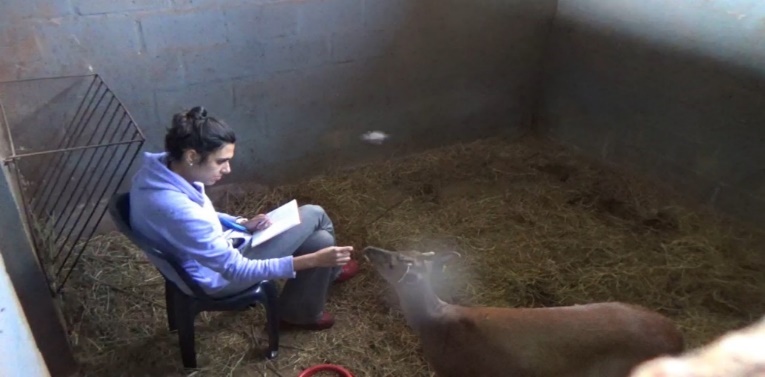


A

B

**S2 fig** . **Illustration of the interaction between a red brocket deer (*Mazama americana*) and the trainer during the learning process.** (**A**) Positive interaction between the trainer and the deer ‘Chico’, during the habituation process. (**B**) Deer ‘Ariel’ is subjected to a desensitization process to spray noise as part of the training protocol.
