# Supplementary material for: Assessing the impact of the reactivity of red brocket deer (Mazama americana) on training efficiency
Source: PLoS One. 2025 Oct 8;20(10):e0315488. doi: 10.1371/journal.pone.0315488 (PMC12507295; doi:10.1371/journal.pone.0315488)
Supplement: S1 Table — (DOCX) [file pone.0315488.s003.docx]

**S2 Table.** **Description and scales of behavioural variables.**

| Variable | Description | Scale |
| --- | --- | --- |
| Approach | Whether the animal approached the unfamiliar person. | Binary (0-1) |
| Restlessness | Restlessness score when in the presence of an unfamiliar person. | Ordinal (1-5) |
| Jump | Whether the animal jumped during the transfer to the handling box. | Binary (0-1) |
| Posture | Posture of the animal inside the handling box. | Ordinal (1-3) |
| Movement | Movement score inside the handling box | Ordinal (1-5) |
